# The Development of Data Collection Tools to Measure Parent–Infant Closeness and Family‐Centered Care in NICUs

**DOI:** 10.1111/wvn.12475

**Published:** 2020-11-19

**Authors:** Anna Axelin, Simo Raiskila, Liisa Lehtonen

**Affiliations:** ^1^ Department of Nursing Science University of Turku Turku Finland; ^2^ Department of Clinical Medicine, Pediatrics Turku University Hospital University of Turku Turku Finland; ^3^ Department of Paediatrics and Adolescent Medicine University of Turku Turku Finland

**Keywords:** data accuracy, data collection methods, family‐centered care, infant closeness, neonatal intensive care unit, parent, skin‐to‐skin contact

## Abstract

**Background:**

Preterm and sick infants benefit from parent–infant closeness and family‐centered care (FCC) in neonatal intensive care units (NICUs). Prospective and feasible tools are needed to measure these care practices to facilitate their implementation.

**Aims:**

To describe the development process of three prospective data collection tools that measure parent–infant closeness and the quality of FCC.

**Methods:**

Data collection tools were developed in an iterative process consisting of three development cycles. Feedback was gathered from parents, staff, and researchers. The first stages of development focused on the content validity, appropriate scaling, and optimization of the response rate of these tools.

**Results:**

The study included parents of 490 infants and the nurses working at bedside in 15 NICUs in six countries. The Parent‐Infant Closeness Diary was developed to measure the daily duration of parental presence, holding, and skin‐to‐skin contact. The optimal duration for daily diaries was 14 consecutive days to maintain a good response rate. Parents provided reliable documentation of parent–infant closeness. Digital FCC tools covering the nine aspects of FCC for parents and nurses were developed to measure the quality of FCC. Participants provided answers on a 7‐point Likert scale. Parents’ response rates remained >50% for approximately 1 month, and the nurses’ mean response rate was 55% (39%–87%) for the 3‐month study period.

**Linking Evidence to Action:**

These new tools provide prospective daily information to aid the implementation of parent–infant closeness and the quality of FCC in NICU in different countries. They can be used to study and evaluate the implementation of these clinical practices NICUs in an international context.

## BACKGROUND

The natural environment for a newborn baby is close to a parent. Parent–infant closeness is a challenge during hospital care, and preterm and sick infants are likely to be exposed to separation during hospitalization (Flacking et al., 2012). In the FCC approach, healthcare professionals work in partnership with parents to promote parent–infant closeness and parent active participation (Mikkelsen & Frederiksen, 2011). Both physical closeness and FCC have improved infants’ growth (Boundy et al., 2016; Lester et al., 2014; O’Brien et al., 2018) and neurobehavioral development (Vohr et al., 2017), as well maternal mental well‐being (Ahlqvist‐Björkroth, Axelin, Korja, & Lehtonen, 2019). This evidence suggests that parent–infant closeness and FCC should be implemented in NICUs to optimize later developmental outcomes of preterm infants (Flacking et al., 2012).

Although parent–infant closeness and FCC are evidence‐based practices, their implementation is a challenge due to differences in NICU facilities and practices regarding parents’ presence and FCC. Earlier research has typically reported the number of parents’ visits in NICUs based on patient charts (Franck & Spencer, 2003; Lester et al., 2014). Only a few studies have documented the duration of parents’ presence or skin‐to‐skin contact (SSC; Blomqvist, Rubertsson, & Nyqvist, 2011; Gonya & Nelin, 2013; Reynolds et al., 2013). In a meta‐analysis on the effects of SSC, only 13% of the 124 controlled studies measured SSC duration (Boundy et al., 2016). Family‐centered care has been reported using retrospective surveys that are usually applied at discharge, which may be burdensome with many questions (Dall'Oglio et al., 2018) and susceptible to biases. Nurses overestimate the level of support they provide to parents; many times, it is not in line with parents’ perceptions and needs (Franck & Axelin, 2013). Interview and observation methods provide more insight into the implementation process but demand a substantial amount of research expertise and resources (Flacking & Dykes, 2013). An obvious need exists for practical and prospective tools to measure parent–infant closeness and the quality of FCC reliably. Such tools are needed for research and to support a change in the care culture (Harvey & Kitson, 2016).

Tool development is a systematic and iterative process aimed at valid and reliable measurement of the target outcome (Rattray & Jones, 2007). The starting point of this process is clearly defining and operationalizing the concept being measured (e.g., physical closeness or FCC), describing what the tool is measuring (e.g., the duration of physical closeness or the quality of FCC), and choosing an appropriate scale. The commonly accepted elements of FCC are respect for individual differences, information sharing, parent autonomy and control, collaboration, negotiation, shared responsibility in infant care, and emotional support for parents (Kuo et al., 2012; Mikkelsen & Frederiksen, 2011). These elements should be used to generate items for tools under development. Instrument development is followed by piloting the questionnaire and evaluating the clarity of the items and the distribution of responses across the scale. After the initial development process, the tool is evaluated for validity and reliability (Rattray & Jones, 2007).

The aim of this article was to describe the first stages of development for prospective data collection tools, including electronic data collection, to measure parent–infant closeness and FCC.

## METHODS AND RESULTS

### Design

The data collection tools were developed using iterative design (Gould & Lewis, 1985). Parents, staff, and the researchers provided deployment feedback that was integrated into short duration, concurrent implementation, and deployment phases comprising a full development cycle. The collected feedback was analyzed during periodic research group meetings and integrated into the continuous development of the tools. Modifications were made to each tool after Cycle 1 of development based on feedback from the parents and nurses. Technical problems that arose were also fixed. The improved data collection tools were tested in Cycle 2, and suggestions for further modifications were noted. Before the data collection tools were used in Cycle 3, modifications were made within a Separation and Closeness Experiences in Neonatal Environment (SCENE) research group (University of Turku, 2018). The modifications are described, in detail, separately for each data collection tool.

### Settings and Participants

Development was conducted in 15 NICUs in Finland, Sweden, Norway, Estonia, Italy, and Spain. The parents of 490 admitted infants participated in the study. Every nurse working at the infants’ bedsides in the participating units was asked to participate during the 3‐month recruitment and data collection periods. The settings and participants are described in more detail in Table S1.

### Translation Process

The process of translating official study material followed Wild et al.’s (2005) 10‐step guideline. Certified translators forward translated the English versions of data collection tools to seven target languages. The responsible researchers in each country reconciled the forward translations to their contexts. After the second certified translators back translated the adapted forward translations to English, the cognitive‐debriefing group discussed the back translations with researchers in each country using Skype.

### Data Collection Tools

#### Parent‐infant closeness diary: content validity and modifications

The initial version of the Parent‐Infant Closeness Diary was developed to measure the time mothers and partners were present in a NICU and the time they spent providing SSC. One diary page was dedicated to the time scales of 1 day, as similarly used in Baby Day Diary (Barr, Kramer, Boisjoly, McVey‐White, & Pless, 1988). The time intervals could be marked with an accuracy of 5 min. In the initial diary, there were four different timelines: mother present, mother SSC, partner present, and partner SSC. Presence in the unit was defined by being inside the unit and not necessarily in the room with the baby all the time, to avoid the burden caused by documenting short interruptions such as visits to the bathroom or coffee breaks. Skin‐to‐skin contact was defined as the infant lying on the parent’s bare chest, dressed only in a diaper and maybe a cap. Parents were asked to fill in the diaries prospectively throughout the time their infants were in a hospital. The diaries were in paper format.

During Cycle 1, the parents gave feedback, both verbally and by free mobile text messages, that they wanted an opportunity to explain the reasons for empty or missing diary days. Based on the feedback, we added space in the diary for parents to explain an empty or missing diary page. During Cycle 2, none of the parents used the opportunity to provide additional information on the extra page provided. For Cycle 3, the page was structured better so that the parents were given tick‐box choices to report the following information regarding the missing diary days: 1. None of the parents were present in the unit; 2. the parents did not remember to fill in the diary; 3. the baby was on “permission at home”; and 4. other important information. In Cycle 3, parents recorded 4,095 diary days. Parents of 86 infants recorded 381 days of missed diary. Reasons for missed entries included the following: Parents were not in the unit for 238 days, the infant was at home for 61 days, the parents forgot to fill in the diary for 60 days, and parents wrote via free text for 22 days.

The interdisciplinary SCENE research group, which included neonatologists, NICU nurses, psychologists, and health and social scientists (*N* = 18), suggested adding a timeline for holding because SSC is rare in some units. For Cycle 3, holding was added in the diary and defined as the baby being in a parent’s arms (i.e., away from the incubator, cot, or bed). The Parent‐Infant Closeness Diary 3rd ed. was published by Raiskila et al. (2017).

##### Response rates over the study period

**Figure 1:**
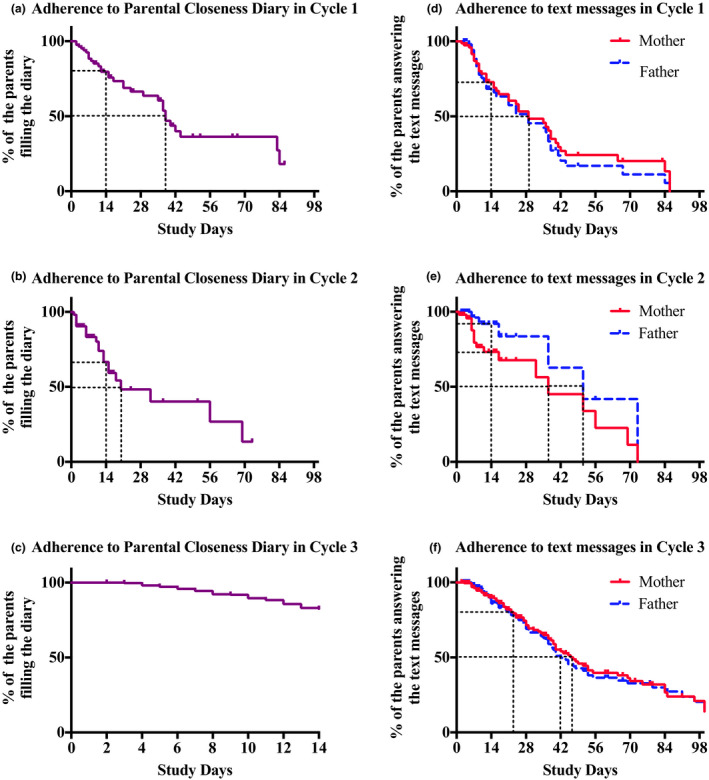
Kaplan–Meier curves showing how long the parents filled in the Parent‐Infant Closeness Diary (a–c) and answered the text message questions (d–f) in Cycle 1 (a, d), Cycle 2 (b, e), and Cycle 3 (c, f).

The number of families participating in each cycle is presented in Table 1. The parents were asked to fill in diaries throughout the hospital stay during Cycles 1 and 2. The response rate clearly declined after 14 days for patients who were still in the hospital (Figure 1). Therefore, the diary period was set to 14 days in Cycle 3 to ensure representative data (Table 1).

**Table 1:** The Iterative Study Cycles of the Development of the Parent–Infant Closeness Diary

Step of tool development	Cycle 1	Cycle 2	Cycle 3
Content validity and modification	Initial version of the diary included parental presence and SSC Diary kept during the entire hospital stay	Free space added to report reasons for missing diary entries	An expert panel added holding. Data sheet for missing diary entries was structured Diary kept only for 14 days plus 7 days at each month of age
Participation and response rate over the study period	89/144 of eligible families participated 80% of the families provided data ≥14 days, 50% of the families ≥38 days	69/114 of eligible families participated 67% of the families provided data ≥14 days, 50% of the families ≥18 days	262/440 of eligible families participated 83% of the families provided data ≥14 days
Reliability	No diary data on 13/446 (3%) of the days for mothers and 48/349 (14%) of the days for partners but text messages indicated presence		No diary data on 6/393 (2%) days for mothers and 20/309 (6%) days for partners but text messages indicated presence SSC data compared to nurses’ documentation: On 57/470 (12%) days, parents reported SSC, but the nurses’ reporting indicated no SSC On 16/470 (3%) days, nurses documented SSC, but parental diary indicated no SSC

##### Reliability

The coverage of diary data was examined with the Finnish data from Turku University Hospital in Cycles 1 and 3. The coverage of diary days was compared with the information received with the tool measuring the quality of FCC. Two to 3% of the mothers’ diary days and 7% to 14% of the partners’ diary days were missing when compared to FCC data indicating presence.

The reliability of the SSC in the parental diaries was examined by comparing diary data to nurses’ documentation during Cycle 3 in Turku University Hospital. Nurses’ documentation missed 12% of the SSC episodes documented in parental diaries. On the other hand, parental diaries missed 3% of the SSC episodes documented by the nurses. The raw agreement was 77%, and the weighted kappa correlation was 0.63.

#### Digital FCC (DigiFCC)‐P for parents to evaluate the quality of FCC: content validity and modifications

Seven text message questions were initially developed based on the FCC literature (Mikkelsen & Frederiksen, 2011; Shields & Tanner, 2004) and the concept of empowerment (Ellis‐Stoll & Popkess‐Vawter, 1998) to measure the following aspects of FCC: 1. parent willingness to participate in infant care; 2. active listening to the parents; 3. parents’ participation in infant care; 4. individualized support by staff; 5. parents’ participation in decision making on infant care; 6. parents’ trust in staff’s infant care; and 7. parents’ participation in medical rounds.

In the initial version of the DigiFCC‐P, one out of the seven text message questions was sent to both the mother and the partner via a protected website every day at 9 p.m. as long as the parents continued to answer the text messages. Responses were rated on a 5‐point Likert scale (1–5, higher scores equated better quality of FCC). Parents chose zero on the scale to indicate they had not been in the unit that day. In addition, parents had the option to write free text in their reply. The questions were randomized for all participating parents in the study unit. To make it easy for the parents to stop receiving questions (during Cycle 1), the parents did not receive any further questions when they did not reply to one text message.

Based on parents’ feedback about their preference not to receive the same question on consecutive days, the randomization of the questions was changed so that each parent received all seven questions in a random order over 7 days. Because some parents had unintentionally dropped out when they had forgotten to answer one question, two reminders (the same question following an unanswered question was sent over 2 days) were added during Cycle 2. This improved the duration of daily responses. However, some parents reported that two reminders were confusing, so the final solution during Cycle 3 was to program the reminder only once, 1 day after the unanswered question.

Before Cycle 3, the interdisciplinary SCENE research group participated in the development of the content validity of the DigiFCC‐P. The question on parents’ willingness to participate in infant care was omitted, and three new questions were added related to parents’ trust in the staff, individualized information based on parents’ needs and background, and emotional support given to parents. In addition, the response scale was changed from a 5‐point to a 7‐point Likert scale to allow more variation in replies. The coefficient of variation increased from 25% to 30% (the difference using the Levene test *p* < .001). The variation ratio for Cycles 1 and 2 was VR = 1 − (*f*_mode_/*N*) = 1 − (1,529/3,023) = .49, and for Cycle 3, it was VR = 1 − (3,654/8,197) = .55. Raiskila et al. (2016) published the Cycle 3 version of the DigiFCC‐P.

##### Response rates over the study period

The number of families participating in each cycle is presented in Table 2. Kaplan–Meier curves showed that the proportion of mothers who replied up to at least 14 days increased after each development cycle. However, the proportion of partners decreased by 14% from Cycle 2 to Cycle 3 (Figure 1). The frequency of parents’ responses to the text messages is shown in Table 2.

**Table 2:** The Iterative Study Cycles of the Development of the DigiFCC Tools Regarding the Quality of FCC

Step of tool development	Cycle 1	Cycle 2	Cycle 3
Content validity and modifications	Seven text message questions and six online questions		One question was omitted, and three new questions were added
	Response on 5‐point Likert scale		Response on 7‐point Likert scale
	Questions randomized within the parents of unit	Questions randomized with the parent	
	No reminders	Two reminder questions	One reminder
Participation and response rate over the study period	63/144 of eligible mothers and 78/144 of eligible partners participated	44/114 of eligible mothers and 63/114 of eligible partners participated	251/440 of eligible mothers and 207/440 of eligible partners participated
	68% of the mothers and 62% of the partners provided data ≥14 days. 50% of the mothers ≥28 days. 50% of partners ≥22 days	76% of the mothers and 88% of the partners provided data ≥14 days. 50% of the mothers ≥43 days. 50% of the partners ≥50 days	83% of the mothers and 74% of the partners provided data ≥14 days. 50% of the mothers ≥37 days. 50% of the partners ≥26 days
	2,239 text messages were sent to the mothers and the partners	962 text messages were sent to the mothers and the partners	9,696 text messages were sent to the mothers and partners
	3,152 answers were received from nurses via online questions	2,429 answers were received from nurses via online questions	11,132 answers were received from nurses via online questions
Mean score, range, and variation of responses in each cycle	*Text messages:* Mean (SD): 4.2 (1.1) Range: 1–5 Variation ratio: 0.49^a^ Coefficient of variation: 25%^a^	*Text messages:* Mean (SD): 4.2 (1.0) Range: 1–5 Variation ratio: 0.49^a^ Coefficient of variation: 25%^a^	*Text messages:* Mean (SD): 5.7 (1.7) Range: 1–7 Variation ratio: 0.55 Coefficient of variation: 30%
	*Online questions:* Mean (SD): 4.1 (0.8) Range: 1–5	*Online questions:* Mean (SD): 4.2 (0.8) Range: 1–5	*Online questions:* Mean (SD): 5.7 (1.5) Range: 1–7

^a^ Cycles 1 and 2 were combined in the calculations of variation ratio and coefficient of variation.

#### DigiFCC‐N for nurses to evaluate the quality of FCC: content validity and modifications

Nurses’ perceptions of the quality of FCC were initially measured using six questions corresponding to the parents’ questions, excluding the question about medical rounds because not all nurses participated in medical rounds during their shifts. Nurses’ responses were initially rated on a 5‐point Likert scale. They chose zero on the scale when they had not worked with parents during their shift. In addition, the nurses had the option to write free text in their reply.

Nurses answered their questions on a website accessible through a research computer in the unit. Every nurse working at bedside was asked to answer one question after each work shift for a 3‐month period. The six questions were randomized in six question blocks. The number of work shifts of nurses working at bedside was collected during the same 3‐month period to calculate the nurse response rate. Because the purpose of the DigiFCC‐N was to evaluate the quality of FCC in the unit level, there was no attempt to match the parents’ and nurses’ answers to each other. Nurses provided one answer representing all families they had worked with during shift. This approach also secured the anonymity of nurses.

Before Cycle 3, the content of the online questions and the scaling was modified analogous to the parents’ text message questions. The mean scores of the nurses’ answers in each cycle are shown in Table 2. Raiskila et al. (2016) published the Cycle 3 version of the DigiFCC‐N.

##### Response rates over the study period

**Figure 2:**
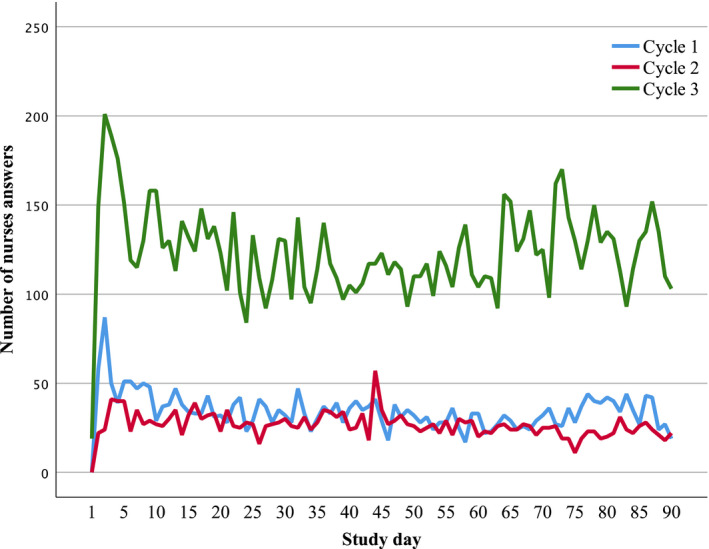
Number of daily answers in nurses’ online questions in Cycles 1 to 3.

In all cycles, the response rate was highest at the beginning and leveled out after a week to about 50% of the initial level (Figure 2). During Cycle 3, the mean coverage of the responses was 55% (39% to 87%) of the work shifts of the bedside nurses (Raiskila et al., 2016). Because one potential source of bias was that one person could give several responses instead of one after her work shift, the responses given within a few seconds were deleted to limit this bias during Cycle 3. A mean of 4.1% (0.1% to 13.3%) of answers was deleted per unit.

## DISCUSSION

Our study reports how we developed and evaluated new data collection tools to measure the duration of parent–infant closeness and the quality of FCC in NICUs. The data collection tools were developed using an iterative design process in collaboration with parents, nurses, and research staff. The tools were shown to be useful and easy‐to‐use in a large international study, and they can be used to support the implementation of parent–infant closeness and FCC.

The optimal duration for daily diaries was 14 consecutive days to maintain a good response rate. Because all parents kept the diary for at least 14 days, if their infants were hospitalized for this time, then the diary method was shown to be feasible and not burdensome for parents. Indeed, parents were a more reliable source of information on SSC compared to nurses’ documentation (Blomqvist et al., 2011).

Our data collection tool provided information on the duration of the parent–infant closeness that is missing in earlier studies (Franck & Spencer, 2003). The duration of closeness emphasizes the time of separation from the child’s perspective and is therefore more meaningful for the infant, as compared to the number of visits. In addition, the duration of parents’ presence and physical closeness is potentially a sensitive outcome measure for interventions such as architectural changes. Shorter parental presence may be the reason for infants’ weaker neurodevelopment in single‐patient room units without a parent’s bed (Pineda et al., 2014) compared to single‐family room units that have improved neurodevelopmental outcomes (Vohr et al., 2017). This tool enables research on the dose–response relationship of parent–infant closeness on child development. In addition, our tool provides information about both parents separately and about the circadian pattern of closeness.

The DigiFCC tools provided reliable, prospective data. This data collection approach felt natural for parents, and one question per day did not burden them too much. Nevertheless, the response rate slowly decreased over time. Although reminders increased the response rates, we limited the number of reminders to one to ensure sensitivity to parents’ wishes to stop answering. The text message questions covered the commonly accepted elements of the FCC concept (Raiskila et al., 2016). A decision was made to focus on the staff–parent relationship and to exclude the social environment outside of hospital or peer support, although those aspects can also be considered elements of FCC. By daily questions from several families, we can obtain a good overall picture of the quality of FCC in a unit on any given day, with little risk of recall bias.

The nurses’ response rate was higher than is usually reported in online questionnaires. In a large sample of studies, a mean response rate was 25 to 30% (Cook, Heath, & Thompson, 2000). The higher response rate could be related to the design, in which only one question per work shift was asked anonymously. The perspectives of parents and nurses provided similar unit profiles (Raiskila et al., 2016), in contrast to earlier studies (Franck & Axelin, 2013). The data were prospective and rooted to the specific day, which is likely to provide more reliable responses than retrospective global ratings.

As limitations, technical problems emerged in the text message questions when the international study was started. The problems were solved because communication network infrastructure has improved. The reliability of SSC episodes was shown to be good in one unit, but further reliability testing would strengthen the method.

## IMPLICATIONS FOR FUTURE RESEARCH

The tools introduced in this paper are useful when studying implementation of parent–infant closeness and FCC in NICUs internationally. These methods provide easy‐to‐use measures to study sustainability of practice changes in a NICU after quality improvement programs. Reliable prospective and quantitative data are needed for the effects of parent–infant closeness and FCC on parent and infant outcomes. Future studies could apply these methods throughout the care episode and beyond, as the transitions are known to be especially vulnerable periods.

The Parent‐Infant Closeness Diary used a paper format. An electronic version of the diary would enable automated data entry and online analysis. In addition to eliminating data entry mistakes, an electronic diary would provide immediate feedback, similar to text message questions, to support the implementation of parent–infant closeness in a unit. In addition, the tools developed in this study require further psychometric testing.

**Linking Evidence to Action**

- Parent–infant closeness and FCC improve later development, especially in preterm infants. Prospective and feasible tools are needed to measure parent–infant closeness and FCC.
- The developed Parent‐Infant Closeness Diary provides daily, prospective, and reliable data on physical parent–infant closeness.
- The DigiFCC tools provide reliable and continuous data on the quality of FCC in a NICU.
- The data collection tools can be used for quality improvement purposes regarding FCC and in research about the effects of parent–infant closeness in NICUs on long‐term outcomes.

## CONCLUSIONS

We developed data collection tools in a collaborative process with parents, staff, and researchers in an international context. The iterative process increased the quality of data collected by these tools. The data collection tools developed in this study can be used to evaluate the implementation of parent–infant closeness and FCC in NICUs in different countries and contexts.

## Supporting information


**Table S1.** The Characteristics of Participating Hospitals and Infants.
