# Supplementary material for: The Development of Data Collection Tools to Measure Parent–Infant Closeness and Family‐Centered Care in NICUs
Source: Worldviews Evid Based Nurs. 2020 Nov 19;17(6):448–56. doi: 10.1111/wvn.12475 (PMC7756210; doi:10.1111/wvn.12475)
Supplement: Supplementary file 1 — Table S1. The Characteristics of Participating Hospitals and Infants. [file WVN-17-448-s001.docx]

**Table S1.** The Characteristics of Participating Hospitals and Infants

|  | **Cycle 1** | | **Cycle 2** | | | **Cycle 3** | |
| --- | --- | --- | --- | --- | --- | --- | --- |
| **Setting** | Four NICUs in Finland (Turku,  Oulu, Lahti, and Pori) in June–August 2012 | | Two NICUs in Finland (Oulu and Vaasa) in June–August 2013;  two NICUs in Finland (Lahti and Pori) in June–August 2014 | | | 11 NICUs in Europe:  Turku, Finland; two NICUs in Stockholm, Sweden; Uppsala, Sweden; Bergen, Norway; Drammen, Norway; Tromsø, Norway; Tallinn, Estonia; Tarto Estonia; Como, Italy; Madrid, Spain | |
| **Participants** | The parents of 92 admitted infants expected to stay for at least 5 days in the NICU; the nurses working at bedside in the units during a 3-month recruitment period | | The parents of 70 admitted infants expected to stay at least 5 days in the NICU; the nurses working at bedside in the unit during a 3-month recruitment period | | | The parents of all inborn infants (*n* = 328) below 35 gestational weeks. Exclusion criteria were (1) the parent not understanding the official language/s of the country, (2) the parent not having a mobile phone, (3) the infants being triplets or a higher order, or (4) the infant being likely to die.  The nurses working at bedside in the unit during a 3-month recruitment period. | |
| **Infant characteristics** | *N* = 92 | | *N* = 70 | | | *N* = 328 | |
|  | *N* | Mean (SD) | *N* | | Mean (SD) | *N* | Mean (SD) |
| Gestational weeks | 92 | 33.7 (4.7) | 70 | 34.7 (4.3) | | 328 | 31.5 (2.9) |
| Birth weight g | 91 | 2,202.6 (1,051) | 69 | 2,461.5 (1,092.3) | | 323 | 1,555.1 (685.7) |
| Birth length cm | 85 | 43.7 (6.1) | 68 | 44.8 (5.4) | | 322 | 40.8 (4.9) |
| Birth head circumf cm | 69 | 30.2 (5.5) | 47 | 31.7 (3.7) | | 318 | 29.2 (3.6) |
|  | *N* | % | *N* | | % | *N* | % |
| Sex (female) | 39 | 42.4 | 34 | 47.9 | | 171 | 52.9 |
| Delivery (vaginal) | 40 | 43.5 | 38 | 53.5 | | 177 | 55.0 |
| Twins | 17 | 18.5 | 17 | 23.9 | | 103 | 32.3 |
| Gestational weeks below 35 | 57 | 62.0 | 39 | 55.7 | | 328 | 100.0 |
